# Biliary Microbiota, Gallstone Disease and Infection with *Opisthorchis felineus*

**DOI:** 10.1371/journal.pntd.0004809

**Published:** 2016-07-22

**Authors:** Irina V. Saltykova, Vjacheslav A. Petrov, Maria D. Logacheva, Polina G. Ivanova, Nikolay V. Merzlikin, Alexey E. Sazonov, Ludmila M. Ogorodova, Paul J. Brindley

**Affiliations:** 1 Central Research Laboratory, Siberian State Medical University, Tomsk, Russian Federation; 2 Laboratory of Catalytic Research, Tomsk State University, Tomsk, Russian Federation; 3 Research Center for Neglected Diseases of Poverty, Department of Microbiology, Immunology and Tropical Medicine, School of Medicine & Health Sciences, George Washington University, Washington, D.C., United States of America; 4 Lomonosov Moscow State University, Faculty of Bioengineering and Bioinformatics, Moscow, Russian Federation; 5 Surgical diseases department of Pediatric faculty, Siberian State Medical University, Tomsk, Russian Federation; 6 Department of Faculty Pediatrics, Siberian State Medical University, Tomsk, Russian Federation; University of Washington School of Public Health, UNITED STATES

## Abstract

**Background:**

There is increasing interest in the microbiome of the hepatobiliary system. This study investigated the influence of infection with the fish-borne liver fluke, *Opisthorchis felineus* on the biliary microbiome of residents of the Tomsk region of western Siberia.

**Methodology/Principal Findings:**

Samples of bile were provided by 56 study participants, half of who were infected with *O*. *felineus*, and all of who were diagnosed with gallstone disease. The microbiota of the bile was investigated using high throughput, Illumina-based sequencing targeting the prokaryotic 16S rRNA gene. About 2,797, discrete phylotypes of prokaryotes were detected. At the level of phylum, bile from participants with opisthorchiasis showed greater numbers of Synergistetes, Spirochaetes, Planctomycetes, TM7 and Verrucomicrobia. Numbers of > 20 phylotypes differed in bile of the *O*. *felineus*-infected compared to non-infected participants, including presence of species of the genera *Mycoplana*, *Cellulosimicrobium*, *Microlunatus* and *Phycicoccus*, and the Archaeans genus, *Halogeometricum*, and increased numbers of *Selenomonas*, *Bacteroides*, *Rothia*, *Leptotrichia*, *Lactobacillus*, *Treponema* and *Klebsiella*.

**Conclusions/Significance:**

Overall, infection with the liver fluke *O*. *felineus* modified the biliary microbiome, increasing abundance of bacterial and archaeal phylotypes.

## Author Summary

The microbiota of the alimentary tract and other sites of the body influences human health. Contrary to popular belief, the bile within the liver is not sterile, and may host a microbiome consisting of diverse species of microbes. The spectrum of microbial species and their numbers within the biliary system may be influenced by disease including infection with pathogens such as parasitic worms and with gallstone disease, liver cancer and other ailments. Here we examined the microbes in the bile of patients from western Siberia, Russia who were concurrently infected with a food-borne parasitic worm, the liver fluke *Opisthorchis felineus*. Infection with this liver fluke is common in western Siberia, as a consequence of dietary preference for undercooked or smoked fresh-water fishes that often carry the larva of the liver fluke. Using high throughput sequencing targeting a conserved bacterial gene and statistical analyses, numerous bacterial species were identified in the bile of the patients. Infection with the liver fluke modified the biliary microbiome, resulting in abundant and diverse species of bacteria and Archaea.

## Introduction

There is increasing interest in the microbiota with respect to diseases of the gastroenterological system [1, 2] including the liver and biliary tree [3, 4]. Many reports have detailed the colorectal/ fecal microbiota, given that samples of feces are readily accessible using non-invasive approaches. Modifications of the gut microbiota have been documented for a number of liver diseases including primary biliary cirrhosis, primary sclerosing cholangitis, cholelithiasis, [4–6]. Moreover, information is becoming available on the microbial composition of the bile during liver disease [6–8]. Conversely, it had long been considered that during good health the bile was sterile or at least that bile was inimical to bacteria [9], with a few reports indicating colonization of the gallbladder and bile as the consequence of reflux of the duodenal contents, blood-borne infection and infection spread through the portal-venous channels [10].

High throughput sequencing of bacterial 16S rDNA genes has provided information on the present of complex microbiota in the bile environment even in absence of biliary tract morbidity. Studies of pigs show that bacteria from the phyla Proteobacteria, Firmicutes and Bacteroidetes populate the gall bladder ecosystem [9]. The investigation of the feces, bile and gallstones from patients diagnosed with cholelithiasis (gallstones) revealed higher bacterial diversity in the biliary system in the comparison with feces; the biliary tract microbiome of gallstone patients includes >100 bacterial OTUs belonging to six bacterial phyla [6]. On the other hand, dysbiosis of biliary microbiome may play key role in the biliary inflammation, supporting the concept that factors that affect bile duct composition can be associated with liver diseases [4]. In this context, findings in hamsters infected with *Opisthorchis viverrini* demonstrated that infection with this liver fluke not only modifies the intestinal microbiota but revealed the presence of >60 phylotypes of nine phyla in the biliary system associated with the parasites [11]. Moreover, infection of hamsters with *O*. *viverrini* positively correlated with increased co-infection with *Helicobacter pylori* and *H*. *bilis*, both in the fecal microbiota and in the biliary tract within the gut of the liver flukes [12].

Liver flukes excrete and secrete mediators [13], altering liver functions that may modify the biliary environment [14] and which, in turn, may modify the composition of the microbiota [12, 15]. Indeed, interactions between liver flukes and the microbiome can be expected to be dynamic and to modify the metabolic responses specific to opisthorchiasis, as known during infection with other helminths [16, 17]. Molecular markers of inflection with the blood fluke *Schistosoma mansoni* infection were found in urine to be primarily linked to changes in gut microflora, energy metabolism and liver function [18], and infection with *Schistosoma haematobium* leads to changes in bacterial pathobionts in the urinary bladder [19]. Other metabolites known to arise from the activities of helminths including catechol estrogens, oxysterols and their adducts involving host cell DNA and other macromolecules likely also influence the ecology of the microbiome [20, 21]. Also, helminth parasites can harbor endosymbiotic microbes, in particular the rickettsia-like bacteria of trematodes [22] and symbiotic *Wolbachia* of filariae [23]. This study investigated the influence of infection with the fish-borne liver fluke *Opisthorchis felineus* on the biliary microbiome, within a background clinical setting of cholelithiasis.

## Materials and Methods

### Study participants; bile samples

The Ethics Committee of the Siberian State Medical University approved this study. All participants provided written informed consent. Participants ranged in age from 40 to 61 years. Prospective participants who had used antibiotics or probiotics within the previous six months were excluded from the study. Fifty-six participants who had been diagnosed with cholelithiasis (gallstone disease) but who were in disease remission provided samples of bile. Gallstone disease had been diagnosed by B-mode ultrasonography. Thirty of these 56 participants were concomitantly diagnosed with infection with the fish-borne liver fluke, *Opisthorchis felineus*, whereas the remainder (26 persons) was not infected with *O*. *felineus* (below). The bile samples were obtained from the study participants during therapeutic intervention for cholelithiasis involving open or laparoscopic cholecystectomy at the 3-d City Tomsk Hospital, Tomsk, western Siberia. During cholecystectomy, 5–10 ml of bile was aspirated from the gallbladder under sterile conditions, three to five ml dispensed into in a sterile tube, and thereafter dispatched immediately to the laboratory. Two ml bile was clarified by centrifugation (10, 000 *g*, 10 min), the supernatant removed, and the pellet was stored at -80°C until processing. Other aliquots of these biles, ~3 ml were subjected to centrifugation at 5,000 *g*, 10 min, after which the pellet was examined for eggs of *O*. *felineus*.

### DNA extraction

The pellet was diluted into Buffer ASL QIAamp Stool Mini Kit (QIAGEN, Hilden, Germany), 25 mg glass beads (0.1 mm diameter) added to the suspension, the mixture vortexed for 10 seconds, and then subjected to bead-beating (Mini-Beadbeater-24, Bio Spec Products Inc) for three minutes. A second bead beating was performed after incubating the suspension at 70°C, after which phenol-chloroform extraction was undertaken to recover genomic DNAs. Subsequently, the DNA was dissolved in 20 μl TE, and DNA yield was measured using a NanoDrop ND-1000 UV spectrophotometer (Nano-Drop Technologies, Wilmington, DE). DNA was aliquoted to perform the PCR to confirm or not infection with *O*. *felineus* (exclusion) and for the 16S rRNA sequence-based survey of biliary prokaryotes. Control DNA extractions in which 100 μl sterile water replaced biliary DNA were undertaken, in order to address laboratory and sequence-based artifacts that can occur with reagents and kits [24].

### Diagnosis of infection with liver flukes

Status of infection liver fluke infection was established by the microscopic examination for eggs of *O*. *felineus* in the material pelleted from several ml of bile and by PCR to identify the presence of DNA of *O*. *felineus* in the pellet. To confirm the infection, we employed a PCR-real time commercial kit for identification of *O*. *felineus* (Medico-biological Union, Novosibirsk, Russia)[25] following the manufacturer’s guidelines. PCR using bile pellet DNA, above, was performed in a thermal cycler (LightCycler 480, Roche).

### Illumina-based sequencing

The DNA samples were used for a 16S rRNA sequence-based survey of bacterial diversity. Amplicons that cover V3 and V4 hypervariable region of 16S rRNA genes (*Escherichia coli* positions 341–805) were generated by PCR with using Primers Next-16S-1st-F 5’- TCG TCG GCA GCG TCA GAT GTG TAT AAG AGA CAG **CCT ACG GGN GGC WGC AG** -3’ and Next-16S-1st-R 5’- GTC TCG TGG GCT CGG AGA TGT GTA TAA GAG ACA G**GA CTA CHV GGG TAT CTA ATC C**-3’. These primers contain gene-specific sequence (bold-face font) and Illumina adapter sequences. The initial PCR cycles were carried out in MJ Mini thermal cycler (MJ Research). The PCR reactions were performed in the following program: initiation enzyme activation at 95°C for 3 min, followed by 25 cycles consisting of denaturation at 95°C for 30 sec, annealing at 55°C for 30 sec and extension at 72°C for 30 sec. After 25 cycles, the reaction was completed with a final extension of 5 min at 72°C. PCR products were recovered by chromatography on Ampure XP beads (Thermo Fisher Scientific) and deployed in a second PCR. The Illumina Nextera XT Index kit (Illumina Inc., San Diego. CA, USA) were used for multiplexing. Two unique indices located on either end of the amplicon were chosen based on the Nextera dual-indexing strategy. To incorporate the indices to the 16S amplicons, PCR reactions were performed on MJ Mini thermal cycler (MJ Research).

Cycling conditions consisted of one cycle of 95°C for 3 min, followed by eight cycles of 95°C for 30 sec, 55°C for 30 sec and 72°C for 30 sec, followed by a final extension cycle of 72°C for 5 min. After purification of PCR-products on Ampure beads (Thermo Fisher Scientific), the concentrations were measured using Qubit technology (Thermo Fisher Scientific). The libraries were sequenced by 2 × 300 bp paired-end sequencing on the MiSeq platform using MiSeq v3 Reagent Kit (Illumina) at the Faculty of Bioengineering and Bioinformatics, Lomonosov Moscow State University.

### Bioinformatics analysis, phylograms

Analysis of the 16S rRNA gene reads was performed using the QIIME (quantitative insight into molecular ecology) pipeline, version 1.9.0 [26]. The Operational Taxonomic Units (OTUs) picking strategy consisted in usage of the open QIIME reference OTU picking algorithm with the OTU-picking method UCLUST [27]. Chimera-checked GreenGenes taxonomy v13.5 was used as the reference base for taxonomic assignment [28]. After taxonomic assignment and demultiplexing, OTUs present only in reagent control samples were subtracted from *O*. *felineus* infected and *O*. *felineus* non-infected groups to eliminate reads due to contamination. Samples with ≥200 counts were included in the analysis. Alpha diversity within and between groups (infected or not-infected with *O*. *felineus*) samples of was calculated in QIIME using Chao1, Shannon and Simpson alpha metrics at depth of 200 sequences per sample. Alpha diversity comparisons were calculated using a two-sample non-parametric *t*-test and 999 Monte Carlo permutations. Beta diversity was investigated by principal components analysis (PCoA) both on non-normalized and normalized (CSS-algorithm [29]) data with the usage of unweighted Unifrac distance and validated with ANOSIM in QIIME. To examine significance of variation among groups at levels of phylum, we used fitZig model, a metagenomeSeq-package in the R statistical environment [29]. Metagenomic prediction was undertaken using the Galaxy-based PICRUSt algorithm [30] against KEGG database, with statistical analysis of variation among groups analyzed using the Mann-Whitney-Wilcoxon test and logistic regression. S1 Table outlines the pipeline.

To visualize genera, we associated each genus with the OTU from GreenGenes DB and prepared a table of the OTU findings that represents all genera identified. The taxonomic tree, which was created during taxonomic classification stage, was pruned with the usage of the genus list by filter_tree.py script in QIIME workflow. Radial phylograms were constructed using FigTree 1.4.2 and MEGA 7 http://www.megasoftware.net/ [31].

## Results

### Study groups, epidemiological characteristics

Patients hospitalized with cholelithiasis and who were diagnosed also with (n = 30) and without (n = 26) infection with *O*. *felineus* participated in the study. These two groups were similar in age and gender, and relatively similar numbers in each group presented with the comorbidities of pancreatitis and infection with hepatitis C virus (Table 1).

**Table 1:** Brief demographic and epidemiological details of the participants of the study, including age in years, gender, infection status for *Opisthorchis felineus*, and other hepatobiliary diseases.

Variable	Infected with *Opisthorchis felineus*	Not-infected with *Opisthorchis felineus*	P (comparison Infected vs non-infected)
**Cholelithiasis**	30	26	
**Median Age (IQR)**	56 (41–60)	53.5 (40–61)	0.74
**Male/Female**	9/21	8/18	1
**Cholelithiasis & pancreatitis**	2	1	1
**Cholelithiasis & hepatitis C virus**	2	1	1
**Samples included for the study: reads > 200**			
**Cholelithiasis**	21	16	
**Median Age (IQR)**	57.5 (42.5–59.5)	55 (41–60)	0.75
**Male/Female**	6/15	5/11	1
**Cholelithiasis & pancreatitis**	1	1	1
**Cholelithiasis & hepatitis C virus**	1	1	1

### Diverse phylotypes comprised the biliary microbiome during cholelithiasis and opisthorchiasis

**Fig 1:**
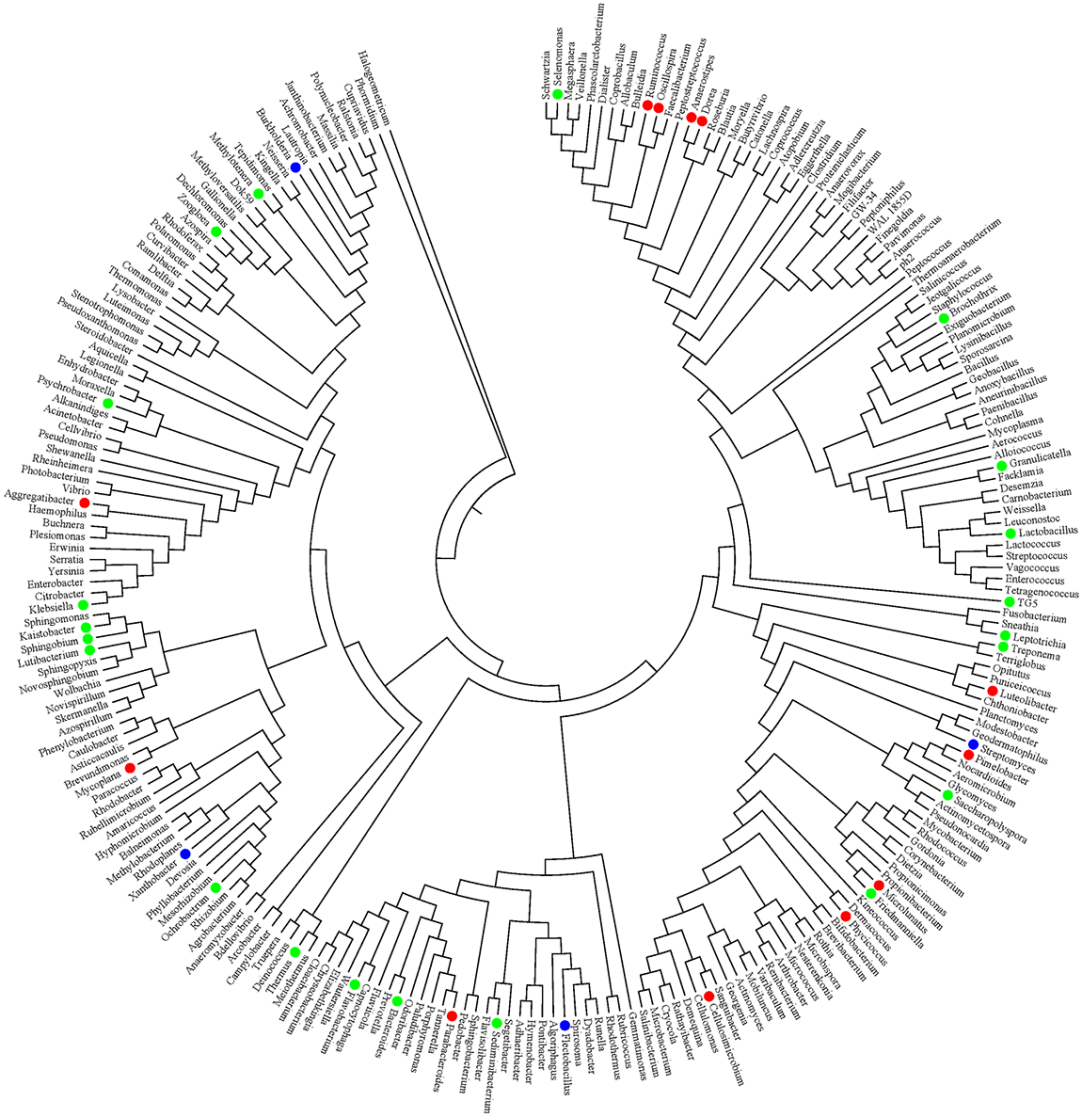
Radial phylogram of 246 genera identified in bile. Red circles indicate phylotypes detected only in the *Opisthorchis felineus* infected group; green colored circles indicate genera that increased in *O*. *felineus* infected group in comparison with the non-infected group, whereas blue circles indicate genera seen in the non-infected participants but not in *O*. *felineus* infected participants.

The Illumina sequencing produced 1,547,628 reads. Demultiplexing showed 628,111 reads were suitable for further analysis. After extraction of reagent contamination controls, there are 81,627 reads and 2,797 discrete OTUs were identified. Taxonomic composition consisted of archaeal and bacterial super-kingdoms, 25 different phyla, 55 classes, 84 orders, 147 families, 246 genera (Fig 1), along with 77 species-level phylotypes that were well supported. Supplementary S2 Table lists several of these latter phylotypes. The median number of reads per sample was 585 (range, 5–10037). However, this wide range in numbers of reads per sample, which spanned two orders of magnitude, hindered comparison among the samples. Accordingly, samples with < 200 reads were not included in the subsequent analysis. After this filtration, reads from the remaining 37 samples were analyzed in depth (Table 1).

### Diversity of the biliary microbiome during opisthorchiasis

**Fig 2:**
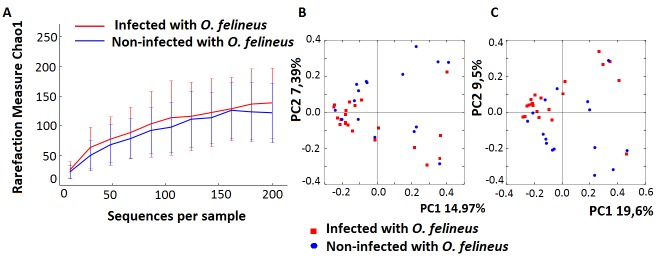
Alpha and beta diversity of the biliary microbiome of 37 study participants. Panel A. Bile content microbiota rarefaction curve generated using Chao1 richness estimator. Samples have been rarified at depth of 200 sequences per sample. Panel B Principal Coordinates Analysis (PCoA) of the bile microbiota in groups infected versus not infected with *O*. *felineus*. Unweighted UniFrac uses non-normalized phylogenetic information to compare samples. ANOSIM was used to evaluate the UniFrac distances of *O*. *felineus* group vs non-infected group (R = 0.087, *p* = 0.038). Panel C. Principal Coordinates Analysis (PCoA) of the bile microbiota in groups infected versus not infected with *O*. *felineus*. Unweighted UniFrac uses phylogenetic information normalized by CSS to compare samples. ANOSIM was used to evaluate the UniFrac distances of *O*. *felineus* group vs non-infected group (R = 0.12, *p* = 0.022).

Alpha diversity was estimated after rarefaction at a depth of 200 sequences per sample by using richness metrics (Chao1, the Shannon and Simpson diversity index) [32]. Analyses of microbial communities did not reveal differences in richness (Chao1 (Fig 2A)), Shannon and Simpson indices (S1 Fig) between participants infected with *O*. *felineus* and non-infected individuals. Principal components analysis (PCoA) of the beta diversity, i.e. community diversity (compositional heterogeneity)/ divergence among samples was undertaken using QIIME, wherein unweighted UniFrac distances ascertained beta diversity. In the case where we used non-normalized phylogenetic data, the first principal coordinate, PC1 accounted for 14.97% of total variance, and after CSS normalization was, PC1 accounted for 19.65% of total variance (Fig 2B and 2C). This difference in bacterial communities between the *O*. *felineus*-infected and uninfected participants was significant, although not robust, and was confirmed using the non-parametric statistical test analysis of similarity (ANOSIM), unweighted Unifrac—R = 0.12, *P* = 0.02 (normalized data). As presented in S2 Fig, hierarchical clustering analysis confirmed these modest differences among the bacterial communities.

To consider the influence of host sex on cholelithiasis [33], we examined the richness metrics (Chao1, the Shannon and Simpson diversity index)after rarefaction at a depth of 200 sequences per sample from the female versus male participants. Chao1 analysis revealed that the diversity was higher in the female in comparison with the male participants (*p* = 0.0461) (S3 Fig).

### Discrete biliary microbiomes during infection with the liver fluke, *O*. *felineus*

**Fig 3:**
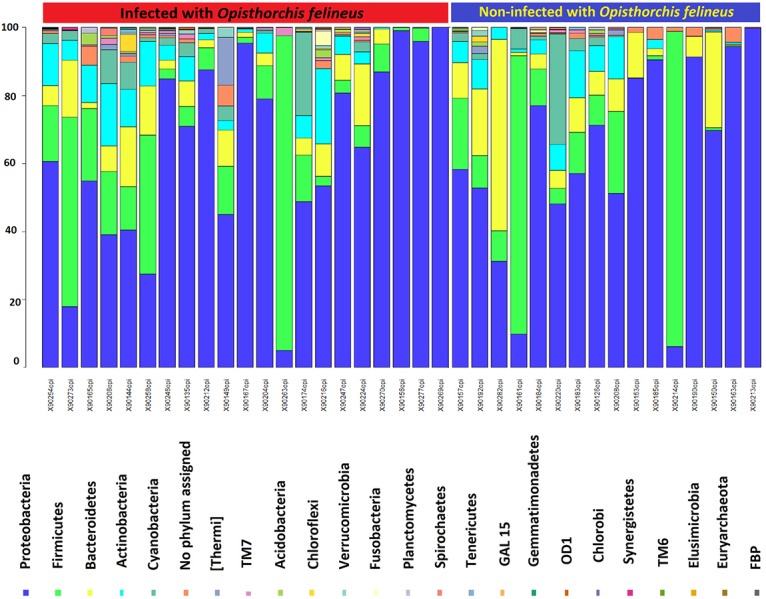
Composition at phylum level of the biliary microbiome. Relative abundances of bacteria (phylum) observed in bile samples.

**Fig 4:**
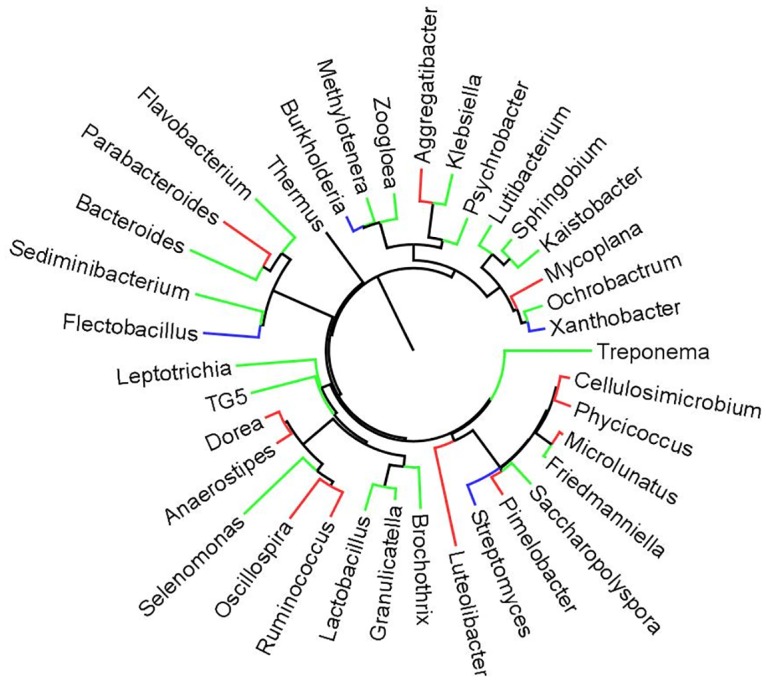
Radial phylogram to display biliary microbiota where groups infected or not with *Opisthorchis felineus* were compared at the genus level. The phylogram displays topology. Genera in red color found only in *O*. *felineus* infected group; genera marked in green increased in the *O*. *felineus*-infected group, in comparison with the non-infected group, whereas blue circles indicate genera seen in the non-infected participants but not in *O*. *felineus* infected participants.

Four phyla, the Proteobacteria, Firmicutes, Bacteroidetes and Actinobacteria dominated the biliary microbiota in the participants of this study, all of whom were diagnosed also with cholelithiasis (Fig 3; S4 Fig). However, the contribution by members of phylum Spirochaetes was significantly increased during infection with *O*. *felineus*. At the level of genus, this was exemplified by increases in *Treponema* (Fig 4; Table 2). Also at the phylum level, higher proportions of Planctomycetes (*P* ≤ 0.01), Synergistetes (*P* ≤ 0.01), Verrucomicrobia (*P* ≤ 0.01), and TM7 (*P* ≤ 0.01) were evident in the group of participants infected with *O*. *felineus* in comparison with the uninfected participants.

**Table 2:** Numerous phylotypes differed between the biliary microbiota of study participants who were positive versus negative for infection with the liver fluke, *Opisthorchis felineus*. The table lists details of 22 phylotypes where numbers of reads counts significantly increased or decreased in relation to liver fluke infection.

Species	Reads *Of* +ve	Reads *Of* -ve	log fold change	Adg p	% of samples that contain reads in *Of*+ group	% of samples that contain reads in *Of*- group	Mean of reads in *Of*+ group	Mean of reads in *Of-* group
Lactobacillus brevis*	4941	4	11,9	<0.0001	4,8	25	235,29	0,25
**Veillonella *dispar***	3	0	0,99	<0.0001	14,3	0	0,14	0
Methylotenera mobilis*	28	1	3,5	<0.0001	9,5	6,25	1,33	0,06
***Paracoccus aminovorans***	6	0	1,2	<0.0001	19	0	0,29	0
Treponema amylovorum*	11	1	2	<0.0001	14,3	6,25	0,52	0,06
***Staphylococcus equorum***	127	14	3	<0.0001	33,3	25	6,05	0,88
***Corynebacterium durum***	19	1	2,5	<0.0001	14,3	6,25	0,90	0,06
***Parabacteroides distasonis*** *	8	0	1,7	<0.0001	14,3	0	0,38	0
***Sphingomonas changbaiensis***	9	0	1,2	<0.0001	28,6	0	0,43	0
***Methylobacterium adhaesivum***	21	5	3,8	<0.0001	4,8	12,5	1	0,31
***Faecalibacterium prausnitzii***	7	1	1,3	0,0002	14,3	6,25	0,33	0,06
***Anoxybacillus kestanbolensis***	9	2	1,47	0,0003	14,3	6,25	0,43	0,13
Bacteroides uniformis*	12	8	2,1	0,0003	9,5	6,25	0,57	0,5
***Pseudoxanthomonas mexicana***	31	8	1,6	0,003	23,8	12,5	1,48	0,5
Sphingobium xenophagum*	31	8	1,7	0,003	19,0	12,5	1,48	0,50
***Haemophilus parainfluenzae***	22	8	1,5	0,004	19,0	12,5	1,05	0,50
***Rathayibacter caricis***	11	5	1,7	0,004	9,5	6,25	0,52	0,31
***Janthinobacterium lividum***	9	5	1,1	0,004	14,3	12,5	0,43	0,31
***Sphingomonas yabuuchiae***	19	10	1,5	0,009	14,3	12,5	0,90	0,63
***Bacillus flexus***	20	10	0,98	0,01	9,5	12,5	0,95	0,63
***Jeotgalicoccus psychrophilus***	2	11	-1,88	0,0002	9,5	12,5	0,10	0,69
Treponema socranskii*	3	9	-2,1	0,002	9,5	6,25	0,14	0,56

* Genera that differed between the groups where participants were infected (*Of* +ve) with or not infected (*Of* -ve) with *O*. *felineus*.

We identified all significant taxa aggregated to OTUs in the bile microbiota associated with the liver fluke infection. Differences were apparent at taxonomic levels from phylum to genus. At the level of genus, 22 phylotypes differed between these two groups. Most phylotypes that differed were detected in higher abundance (i.e. absolute read counts) in bile from the *O*. *felineus*-infected participants (Fig 4; Table 2). Among specific examples, there was elevated abundance of *Klebsiella* spp., *Aggregatibacter* spp., *Lactobacillus* spp., *Treponema spp*., *Haemophilus parainfluenzae* and *Staphylococcus equorum* in bile of participants infected with liver flukes. In addition, *Veillonella dispar*, *Paracoccus aminovorans*, *Parabacteroides distasonis*, *Sphingomonas changbaiensis*, *Cellulosimicrobium* sp., *Phycicoccus* sp. and others were detected solely in bile from persons infected with *O*. *felineus* (Fig 4; Table 2), whereas *Flectobacillus* sp., *Xanthobacter* sp., *Burkholderia* sp., *Streptomyces* sp., *Jeotgalicoccus psychrophilus and Treponema socranskii* increased in the uninfected group vs the group with infection with *O*. *felineus* (Table 2). Reads assigned to the super-kingdom Archaea were identified in the microbial community of bile from one of the *O*. *felineus*-infected persons; these reads aggregated with a phylotype from the Phylum Euryarcheota, genus *Halogeometricum*.

Given the potential for pathogenic microbes for involvement in cholelithiasis [6, 34], a list of phylotypes identified in bile samples is presented. Also, we searched the list of phylotypes for the presence of bacteria that had been described as associated with the human biliary tract by Shen and coworkers [7]. We compared the list of phylotypes detected in the present study in the bile of participants presenting with gallstone disease (37 individuals) with the list of microbes recently described in human gallstones and bile [7]. About 9% of the same species were identified here, including *Rothia aeria*, *Haemophilus influenza*, *Veillonella dispar*, *Acinetobacter johnsonii*, *Acinetobacter lwoffii* and *Streptococcus anginosus* (S2 Table).

The prediction of functional KEGG pathway abundances from the 16S rDNA-based metagenomes was accomplished using PICRUSt. The same predicted functional pathways characterized the *O*. *felineus* infected and *O*. *felineus* non-infected bile, so that functional differences were not evident. Predicted metagenomes at the three hierarchical KEGG pathway levels revealed the functional categories represented in the bile microbiota of patients with cholelithiasis. Membrane transport, carbohydrate metabolism and amino acid accounted for more than one third of the hypothetical functions from the KEGG pathways at level 2 (S3 Table).

### Database accession

Sequence data obtained have been deposited to the European Nucleotide Archive, accession number PRJEB12755, http://www.ebi.ac.uk/ena/data/view/PRJEB12755.

## Discussion

Although it had been assumed that the biliary system in a healthy person is a sterile organ, it is now apparent that bile supports a complex microbiome in otherwise healthy individuals [4, 9]. Nonetheless it has long been known that cholelithiasis, cholecystitis and cholangitis lead to bacteriobilia [35, 36]. The presence of bacteria in the bile and gallbladder/gallstones has been diagnosed by microbial culture, where positive culture of bile during cholelithiasis and chronic cholecystitis ranges from 0–81% [37, 38]. Frequently identified are *Escherichia coli*, and species of *Enterococcus*, *Klebsiella*, and *Pseudomonas* [38–40].

Analysis by pyro-sequencing targeting the bacterial 16S rRNA gene revealed that phylotypes of the phylum Firmicutes were dominate the bile of healthy pigs, with Proteobacteria and Actinobacteria also prominent, and with lesser contributions from other phyla. Firmicutes, Proteobacteria and Bacterioidetes, dominate the human biliary microbiome of gallstones and bile during cholelithiasis [6]. Our present findings accord with these reports [6]. Biliary tract microbiota of participants with cholelithiasis showed substantial person-to-person variation; the relative abundance of phylum Firmicutes varies 0–92% through the different samples. Similar phenomena have been reported for microbiota of gallstones from residents of Kunming, China [7]. Nonetheless, species contributing to biliary microbiota of the participants from Siberia differed markedly from microbes reported form China. Phylotypes previously identified in bile also were present, including and *Haemophilus parainfluenzae*, *Enterobacter cloacae* [41, 42], and *Streptococcus anginosus*, which is associated with pyogenic liver abscess [43]. In addition, microbes associated with periodontal disease, including *Treponema socranskii* [44], *T*. *amylovorum* [45], *Veillonella dispar*, [46], *Aggregatibacter segnis* [47], and *Bacteroides eggerthii* [48] were identified. Others more usually known from the external environment, including soil, plants, and rivers, also were identified including *Sphingomonas changbaiensis*, *Rathayibacter caricis*, *Bacillus flexus*, *Methylobacterium adhaesivum*, *Psychrobacter pacificensis*, and *Pseudomonas umsongensis*.

Although alpha diversity of the biliary microbiome did not appear to be impacted during infection with *O*. *felineus*. A diverse often contradictory literature has accumulated over past decade on the influence of helminth infection on the microbial diversity of the intestines. Among other examples, polyparasitsm by soil-transmitted nematodes (*Ascaris*, *Tichuris*, hookworms) results in increased diversity of gut microbiota in indigenous Malaysians and microbial diversity decreases following deworming [49]. By contrast, in other situations, increasing alpha diversity is not apparent during trichuriasis [50]. In comparison, infection with *O*. *felineus* lead to the modification of composition of the bile microbiome. Specifically, most of the phylotypes that differed were detected in higher abundance in bile during opisthorchiasis although some phylotypes decreased; *Jeotgalicoccus psychrophilus*, a Gram-positive halophile [51, 52] was included among the latter. *Lactobacillus* spp. increased in richness in *O*. *felineus*-infected bile. Colonization of the gut by nematodes has been shown to be associated with increasing prominence of Lactobacillaceae. Mice parasitized by the intestinal nematode Heligmosomoides polygyrus exhibit increased numbers of Lactobacillaceae in the ileum [53] and in the duodenum [54]. Chronic infection of mice with the whipworm *Trichuris muris* also increases the abundance of *Lactobacillus* spp. [55], and similarly hamsters infected with O. viverrini-infected exhibit more *Lactobacillus* in the colon [15]. Intriguingly, *Lactobacillus* species may contribute probiotic defense against allergies [56, 57]. In regions endemic for opisthorchiasis felinea, specifically in western Siberia, liver fluke infection modifies genetic risk of atopic bronchial asthma [58]. Furthermore, in urban regions, the presence of antibodies to O. felineus negatively correlates with the atopic sensitization [59]. There is evidence that the modification of the microbiota by helminths contributes to modulation of allergic inflammation [60, 61]. Our data provided additional support that helminth infection promotes the increase in numbers of *Lactobacillus* species that, in turn, influences the paradoxical relationship between allergic diseases and helminthiasis.

*Haemophilus parainfluenzae* also increased in *O*. *felineus* infected samples; this pathogen is associated with liver abscess [62], and liver abscess represents a serious complication of opisthorchiasis felinea [63]. *Veillonella dispar*, *Paracoccus aminovorans*, *Parabacteroides distasonis*, *Sphingomonas changbaiensis*, among others, were constituents of the biliary microbiome of the liver fluke-positive participants. Although *V*. *dispar* is known from bile [53], *P*. *distasonis* has been described from feces as a risk factor for obesity [64]. These two phylotypes represent microbes typically seen in the human alimentary tract. By contrast, S. *changbaiensis* is known from forest soils [12] and *Paracoccus aminovorans* associates with the skin of fish [65] [66]. In addition, we identified reads that aggregated with the archaeal genus *Halogeometricum* (phylum Euryarcheota). Flesh of salted, dried river fishes represents a dietary stable in regions of Siberia [67]. We speculate that *Halogeometricum* and *Paracoccus aminovorans* may have been transported to the biliary tract with ingested dried fish and/or other fish products contaminated with metacercarie of *O*. *felineus*. Other phylotypes of the Euryarcheota occur in bile of hamsters infected with metacercariae of *O*. *viverrini* [15]. Conveyance of these environmental microbes from the outside world to the human alimentary tract may have been accomplished during establishment of infection by the liver flukes.

Notwithstanding the novelty and complexity of the findings, our study has limitations. The findings associated with *O*. *felineus* took place in the setting of concomitant gallstone disease. The microbial profile of the bile may differ in the absence of cholelithiasis, and furthermore, the pH of the bile (which was not measured here) may have influenced the microbiome [68, 69]. Metabolic changes associated with gallstone formation can lead to microflora discrete from that of healthy individuals [6]. Moreover, we cannot exclude that participants in the non-liver fluke infected cohort had not previously been infected given elevated prevalence of opisthorchiasis felinea in the Tomsk region [70]. Nonetheless, these findings appear to be novel in the context of the biliary microbiome during opisthorchiasis. It will be informative to investigate this phenomenon further, including in people without gallstone disease living in regions where liver flukes are endemic and infection with which represents increased risk for bile duct cancer.

## Supporting Information

S1 Table Pipeline employed for bioinformatics analysis. (DOCX)

S2 Table List of reads for species detected in the bile samples of the participants. (XLSX)

S3 Table List of KEGG pathway abundances from the 16S rDNA-based metagenomes for *O*. *felineus* infected and *O*. *felineus* non-infected groups. (XLSX)

S1 Fig Alpha diversity of the biliary microbiome for the 37 participants. **A** Shannon index after rarefaction at depth of 200 sequences per sample in the *O*. *felineus* infected group vs non-infected group **B** Simpson index after rarefaction at depth of 200 sequences per sample in *O*. *felineus* infected group vs non-infected group.
(DOCX)

S2 Fig Hierarchical clustering of biliary microbiome. Each column of the heatmap corresponds to a bile sample, and each row to a phylum of the Prokaryota identified in the sequence data. The *O*. *felineus* infection status is indicated for each participant by blue (–) or red (+). Lighter colors along the black/yellow spectrum indicate a higher abundance.
(DOCX)

S3 Fig Alpha diversity for the 37 participants. Bile content microbiota rarefaction curve generated using Chao1 richness estimator. **A** Chao1 after rarefaction at depth of 200 sequences per sample in female group and male group, **B** Median± (IQR) of Chao1 in females and males (*p* = 0.0461).
(DOCX)

S4 Fig Bubble plot. Diameter and volumes reveal abundance of phylotypes (after log-transformation) at the phylum level, in bile from 37 study participants.
(DOCX)
