# Supplementary material for: Biliary Microbiota, Gallstone Disease and Infection with Opisthorchis felineus
Source: PLoS Negl Trop Dis. 2016 Jul 22;10(7):e0004809. doi: 10.1371/journal.pntd.0004809 (PMC4957795; doi:10.1371/journal.pntd.0004809)
Supplement: S1 Table — (DOCX) [file pntd.0004809.s001.docx]

**Supplementary Table S1.** Pipeline employed for bioinformatics analysis.

| Step | Command | Note |
| --- | --- | --- |
| Implemented in QIIME | | |
| Joining paired-end MiSeq reads | join_paired_ends.py | Using SeqPrep algorithm |
| Demultiplexing and quality filtering | split_libraries_fastq.py | Phred quality score 19 |
| OTU picking strategy | pick_open_reference_otus.py | OTU-picking method UCLUST, taxonomy - GreenGenes v13.5 database |
| Negative OTUs elimination | filter_otus_from_otu_table.py | Exclusion of all OTUs associated with negative samples |
| Samples summary | biom summarize-table | Samples with less than 200 counts were excluded |
| Individual samples alpha diversity | alpha_rarefaction.py then  add_alpha_to_mapping_file.py | Using Shannon, chao1 and Simpson index. Rarefaction implemented at depth of 200 sequences per sample |
| Alpha diversity between samples | alpha_rarefaction.py then  compare_alpha_diversity.py | Comparison with the usage of non-parametric t-test |
| Beta diversity | beta_diversity_through_plots.py then make_2d_plots.py | unweighted Unifrac distance |
| Beta diversity with data normalization | filter_otus_from_otu_table.py, then  normalize_table.py, then  beta_diversity_through_plots.py and make_2d_plots.py | Include all features that are at least in 3 samples. Normalization with CSS algorithm; unweighted Unifrac distance |
| ANOSIM | compare_categories.py | Number of permutations: 999 |
| Implemented in Galaxy | | |
| Normalization | Normalize By Copy Number |  |
| Metagenome predictions | Predict Metagenome |  |
| Function annotation | Categorize by function |  |
| Implemented in R | | |
| Construction of MRobject | newMRexperiment | MetagenomeSeq-package |
| Filtering | filterData | MetagenomeSeq-package;  Include all features which are at least in 3 samples |
| Normalization | cumNorm | MetagenomeSeq-package (CSS normalization algorithm);  normalization factor = 0.5 |
| Searching of differences in taxonomy between groups | aggTax  fitZig | Taxonomic aggregation was performed on each level |
| Heatmap construction | plotMRheatmap | Heatmap on phylum level |
| Bubble-plot construction | ggplot | Bubble-plot on phylum level |
| Searching of differences in functional annotations (KEGG) between groups | wilcox.test  glm | logistic regression was used for glm model |

.
