# Supplementary material for: Biliary Microbiota, Gallstone Disease and Infection with Opisthorchis felineus
Source: PLoS Negl Trop Dis. 2016 Jul 22;10(7):e0004809. doi: 10.1371/journal.pntd.0004809 (PMC4957795; doi:10.1371/journal.pntd.0004809)
Supplement: S1 Fig — A Shannon index after rarefaction at depth of 200 sequences per sample in the O. felineus infected group vs non-infected group B Simpson index after rarefaction at depth of 200 sequences per sample in O. felineus infected group vs non-infected group. (DOCX) [file pntd.0004809.s004.docx]

**
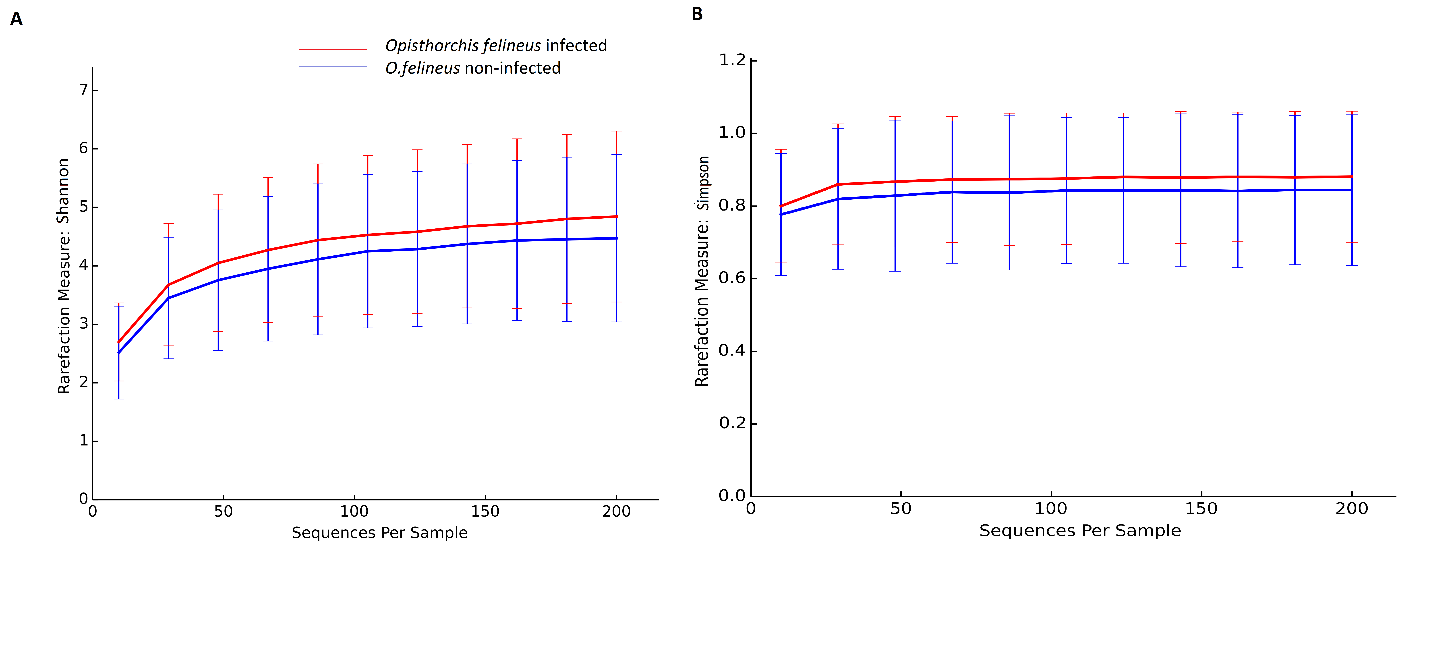
**

**Supplementary Figure S1.** Alpha diversity of the biliary microbiome for the 37 particpnats. **A** Shannon index after rarefaction at depth of 200 sequences per sample in the *O. felineus* infected group vs non-infected group **B** Simpson index after rarefaction at depth of 200 sequences per sample in *O. felineus* infected group vs non-infected group
