# Supplementary material for: Biliary Microbiota, Gallstone Disease and Infection with Opisthorchis felineus
Source: PLoS Negl Trop Dis. 2016 Jul 22;10(7):e0004809. doi: 10.1371/journal.pntd.0004809 (PMC4957795; doi:10.1371/journal.pntd.0004809)
Supplement: S2 Fig — Each column of the heatmap corresponds to a bile sample, and each row to a phylum of the Prokaryota identified in the sequence data. The O. felineus infection status is indicated for each participant by blue (–) or red (+). Lighter colors along the black/yellow spectrum indicate a higher abundance. (DOCX) [file pntd.0004809.s005.docx]

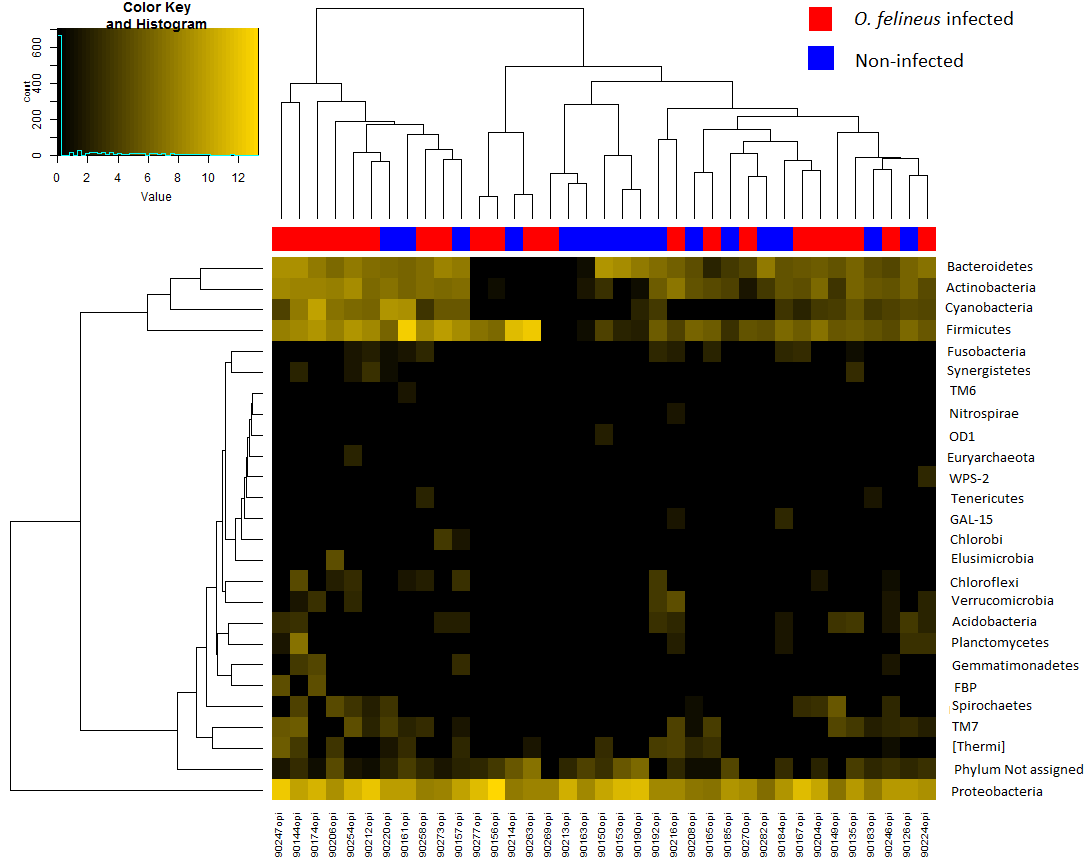


**Supplementary Figure S2**. Hierarchical clustering of biliary microbiome. Each column of the heatmap corresponds to a bile sample, and each row to a phylum of the Prokaryota identified in the sequence data. The *O. felineus* infection status is indicated for each participant by blue (–) or red (+). Lighter colors along the black/yellow spectrum indicate a higher abundance.
