# Supplementary material for: Biliary Microbiota, Gallstone Disease and Infection with Opisthorchis felineus
Source: PLoS Negl Trop Dis. 2016 Jul 22;10(7):e0004809. doi: 10.1371/journal.pntd.0004809 (PMC4957795; doi:10.1371/journal.pntd.0004809)
Supplement: S3 Fig — Bile content microbiota rarefaction curve generated using Chao1 richness estimator. A Chao1 after rarefaction at depth of 200 sequences per sample in female group and male group, B Median± (IQR) of Chao1 in females and males (p = 0.0461). (DOCX) [file pntd.0004809.s006.docx]

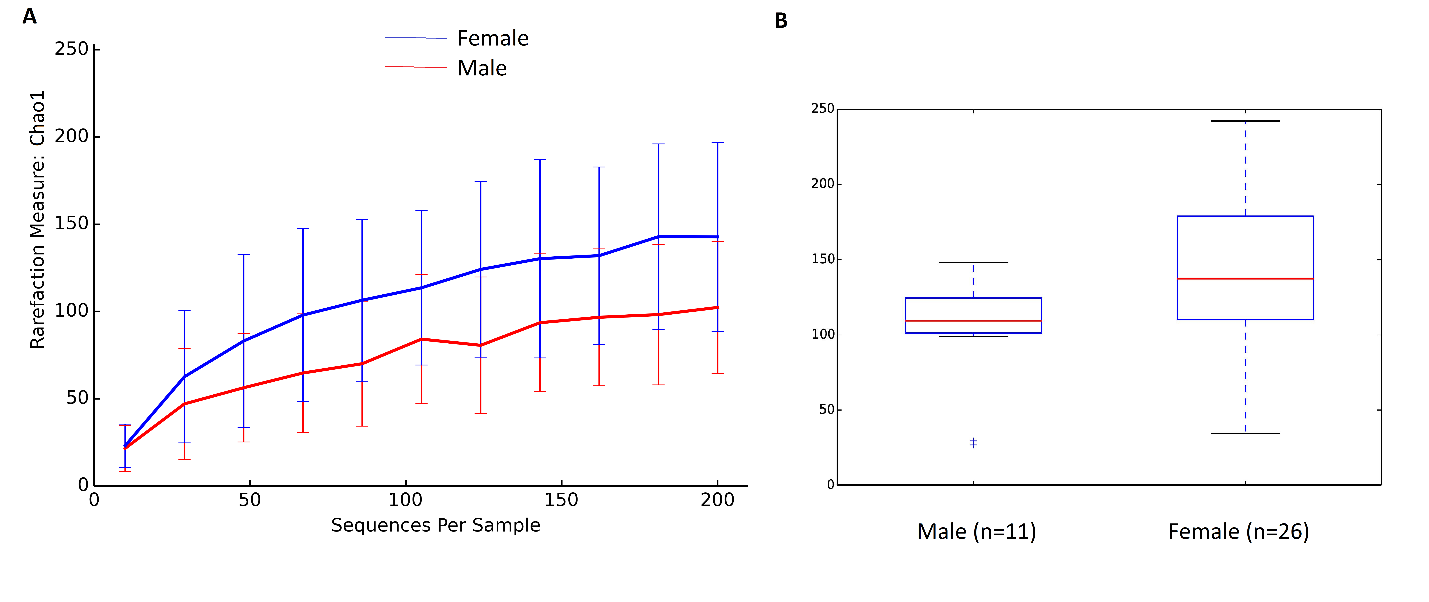


**Supplementary Figure S3**. Alpha diversity for the 37 participants. Bile content microbiota rarefaction curve generated using Chao1 richness estimator. **A** Chao1 after rarefaction at depth of 200 sequences per sample in female group and male group, **B** Median+ (IQR) of Chao1 in females and males (*p* = 0.0461).
