# Supplementary material for: Biliary Microbiota, Gallstone Disease and Infection with Opisthorchis felineus
Source: PLoS Negl Trop Dis. 2016 Jul 22;10(7):e0004809. doi: 10.1371/journal.pntd.0004809 (PMC4957795; doi:10.1371/journal.pntd.0004809)
Supplement: S4 Fig — Diameter and volumes reveal abundance of phylotypes (after log-transformation) at the phylum level, in bile from 37 study participants. (DOCX) [file pntd.0004809.s007.docx]

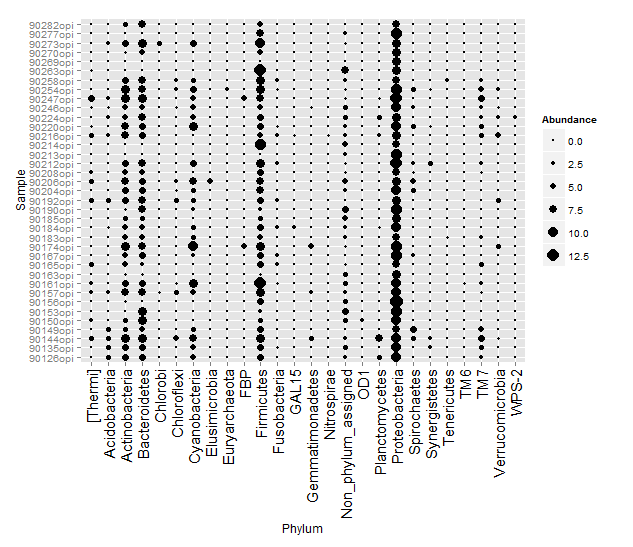


**Supplementary Figure S4**. Bubble plot. Diameter and volumes reveal abundance of phylotypes (after log-transformation) at the phylum level, in bile from 37 study participants.
